# Evaluation of the imputation performance of the program IMPUTE in an admixed sample from Mexico City using several model designs

**DOI:** 10.1186/1755-8794-5-12

**Published:** 2012-05-01

**Authors:** S Krithika, Adán Valladares-Salgado, Jesus Peralta, Jorge Escobedo-de La Peña, Jesus Kumate-Rodríguez, Miguel Cruz, Esteban J Parra

**Affiliations:** 1 Department of Anthropology, University of Toronto at Mississauga, 3359 Mississauga Road North, Mississauga, ON, Canada; 2 Unidad de Investigacion Medica en Bioquimica, Hospital de Especialidades, Centro Medico Nacional Siglo XXI, IMSS, Av. Cuauhtemoc 330, Col. Doctores, C.P. 06720, Mexico City, Mexico; 3 Unidad de Investigacion en Epidemiologia Clinica, Hospital General Regional 1, Dr Carlos McGregor, IMSS, Mexico City, Mexico; 4 Fundacion IMSS, Mexico City, Mexico

## Abstract

**Background:**

We explored the imputation performance of the program IMPUTE in an admixed sample from Mexico City. The following issues were evaluated: (a) the impact of different reference panels (HapMap vs. 1000 Genomes) on imputation; (b) potential differences in imputation performance between single-step vs. two-step (phasing and imputation) approaches; (c) the effect of different INFO score thresholds on imputation performance and (d) imputation performance in common vs. rare markers.

**Methods:**

The sample from Mexico City comprised 1,310 individuals genotyped with the Affymetrix 5.0 array. We randomly masked 5% of the markers directly genotyped on chromosome 12 (n = 1,046) and compared the imputed genotypes with the microarray genotype calls. Imputation was carried out with the program IMPUTE. The concordance rates between the imputed and observed genotypes were used as a measure of imputation accuracy and the proportion of non-missing genotypes as a measure of imputation efficacy.

**Results:**

The single-step imputation approach produced slightly higher concordance rates than the two-step strategy (99.1% vs. 98.4% when using the HapMap phase II combined panel), but at the expense of a lower proportion of non-missing genotypes (85.5% vs. 90.1%). The 1,000 Genomes reference sample produced similar concordance rates to the HapMap phase II panel (98.4% for both datasets, using the two-step strategy). However, the 1000 Genomes reference sample increased substantially the proportion of non-missing genotypes (94.7% vs. 90.1%). Rare variants (<1%) had lower imputation accuracy and efficacy than common markers.

**Conclusions:**

The program IMPUTE had an excellent imputation performance for common alleles in an admixed sample from Mexico City, which has primarily Native American (62%) and European (33%) contributions. Genotype concordances were higher than 98.4% using all the imputation strategies, in spite of the fact that no Native American samples are present in the HapMap and 1000 Genomes reference panels. The best balance of imputation accuracy and efficiency was obtained with the 1,000 Genomes panel. Rare variants were not captured effectively by any of the available panels, emphasizing the need to be cautious in the interpretation of association results for imputed rare variants.

## Background

Genome-wide association studies (GWAS) are a convenient and powerful tool for the identification of common genetic variants associated with complex diseases [1-5]. In recent years, high-density GWAS have proven successful in identifying loci predisposing to a variety of complex diseases, e.g., type 1 and type 2 diabetes, obesity, inflammatory bowel disease, prostate cancer and breast cancer [5,6]. The recent successes of GWAS have mainly been possible due to the rapid advancement in high-throughput SNP genotyping technologies (e.g., Affymetrix and Illumina platforms), which assay a large number of SNPs (between 100,000 and 2,500,000) across the human genome [7-9]. However, despite recent improvements, the coverage of most of the genotyping platforms remains relatively inadequate, in comparison with the total number of SNPs described in the genome. Furthermore, rare variants are typically not included in these genotyping arrays and a fraction of the typed SNPs are eliminated from further analyses, due to genotyping problems, leading to the loss of statistical power in association studies [10-13].

To overcome the aforementioned limitations of GWAS genotyping platforms, a variety of imputation methods have been developed. These methods infer missing or untyped SNP genotypes based on the genotypes at nearby typed SNPs, using the pattern of linkage disequilibrium (LD) observed in reference samples. Imputation methods have been extensively used to predict the genotypes of untyped markers by combining reference panels of individuals genotyped at a dense set of SNPs with a study sample genotyped at a subset of the SNPs [14,15]. The main challenge of imputation, however, lies in the selection of an appropriate reference panel relevant for the study populations. Although this is straightforward in samples with ancestry matching that of the available reference panels (e.g., European or East Asian ancestry), this is not the case for samples that are not well represented in the reference panels (e.g. Native American samples or admixed samples). One of the proposed solutions to the latter scenario is to include mixtures of the available reference panels for imputation. It has been described that this strategy results in good imputation accuracy [16].

The application of imputation methods is cost effective, increases the power and coverage of the study, facilitates meta-analysis, enables combination of data across multiple genotyping platforms, and aids in replication of significant findings [15,17,18]. Several imputation methods are currently available, based on different statistical models. Commonly used imputation programs are IMPUTE [19,20], MACH [21], BEAGLE [22], fastPHASE [23] and PLINK [24]. The relative performance of these programs has been assessed in various studies [19,25-29].

In the present study, we employed the HapMap and the recently available 1000 Genomes reference panels to evaluate the performance of the imputation program IMPUTE in an admixed sample from Mexico City. The following issues were evaluated in this project: (a) the impact of different reference panels (HapMap and 1000 Genomes) on imputation; (b) potential differences in imputation performance between single-step vs. two-step (phasing and imputation) approaches; (c) the impact of different INFO score thresholds on imputation performance and (d) imputation performance in common vs. rare markers.

## Methods

### Study participants and Genotyping

A total of 1,310 individuals from Mexico City (967 with type 2 diabetes and 343 with normal glucose tolerance) were analyzed in this study. Informed consent was obtained from each participant, and the research was approved by the ethical research boards of the Medical Center ‘Siglo XXI’ and the University of Toronto. Genotyping of the sample was then carried out in the microarray analysis facility located in the Centre for Applied Genomics (Toronto, ON, Canada), using the Affymetrix Genome-wide Human SNP array 5.0 (Affymetrix, Santa Clara, CA, USA), and following standard protocols. Further details about participant recruitment and quality control measures can be found elsewhere [30].

### Reference panels for imputation

The following reference panels were used for the present study:

(a) HapMap phase II combined sample, which includes up to 4 million SNPs typed in 269 individuals belonging to East Asian/European/West African ancestry,

(b) HapMap phase II combined sample along with the HapMap phase III Mexican-American LA sample (MXL), which was genotyped for about 1.4 million SNPs, and the

(c) 1000 Genomes phase I sample (June 2011 release), which comprises >37 million autosomal SNPs typed in 1,094 individuals from populations around the world (more information is available at http://www.1000genomes.org/).

### Imputation using IMPUTE

The programs IMPUTE v1 and v2 were employed for imputation of untyped markers. IMPUTE v1 [19] was used for analysis with the HapMap phase II combined and the HapMap phase II combined + HapMap phase III Mexican-American reference datasets and IMPUTE v2 [20] was used with the HapMap Phase II combined and the 1000 Genomes Phase I (June 2011 release) reference panels. With IMPUTE v1 we performed phasing and imputation in a single analytical step. With IMPUTE v2 we used a two-step approach, phasing the study sample first and performing imputation using the reference samples in a second stage.

In order to evaluate the performance of the imputation, we randomly masked 5% of the markers directly genotyped on chromosome 12 (n = 1,046) and compared the imputed genotypes with the Affymetrix genome-wide Human SNP array 5.0 genotype calls. For analysis using IMPUTE v1, chromosome 12 was divided into chunks of 15 Mb length (chunk size specified using the *-int* option). Each chunk was then directly imputed with the following settings: buffer = 250 kb, k = 40, iter = 30, burnin = 10, Ne = 11418, using the said reference panels. The *–buffer* option helps to avoid edge effects when imputing in relatively small chunks.

For analysis using IMPUTE v2, chromosome 12 was broken into smaller chunks of ~5 Mb each, and we also used a buffer region of 250 kb. Phasing of GWAS data in each chunk was subsequently performed to produce the best-guess haplotypes (using *–phase* and –*include_buffer_in_output* flags with IMPUTE v2’s settings: k = 80, iter = 30, burnin = 10, Ne = 11500). Imputation from the best-guess haplotypes was then carried out, for each chunk, using the aforementioned reference panels. The differences in program versions and imputation settings between the one-step and two-step approaches are primarily due to the fact that the imputations were done at different times.

### Evaluation of imputation performance

We report the concordance rate between the imputed and observed genotypes for the masked SNPs as a measure of imputation accuracy and the proportion of non-missing genotypes under a given INFO score threshold as a measure of the imputation efficacy. The program Gtool (http://www.well.ox.ac.uk/~cfreeman/software/gwas/gtool.html) was used for this purpose, using the default INFO score threshold value of 0.9 to export the IMPUTE data to PLINK format. With this threshold, imputed markers with INFO scores < 0.9 were labeled as missing genotypes. Then, the PLINK’s *--merge* command along with *--merge-mode 7* command was used to evaluate the genotype concordance. We also used PLINK to obtain information on the proportion of non-missing genotypes for each of the four imputation strategies(Impute v1: Hapmap phase II combined and Hapmap phase II combined + MXL, Impute v2: Hapmap phase II combined and 1000 Genomes).

We also evaluated the imputation performance (accuracy and efficacy) at different INFO score threshold values (0.8, 0.7, 0.6 and 0.5), in addition to the default threshold value of 0.9. This analysis was carried out only for the two-step imputation method based on the HapMap phase II combined reference sample.

Finally, we explored the effect of allele frequency on imputation performance. The INFO scores based on the two-step imputation method using the HapMap phase II combined and the 1000 Genomes (June 2011 release) reference panels were compared for different allele frequency categories, grouping markers in 5% bins. Histograms were generated to show the distribution of the INFO scores for each bin and the distribution of the differences in INFO scores, and we estimated the correlation between the INFO scores for the two imputation approaches. We also did a more detailed analysis of imputation accuracy and efficacy for markers in the following allele frequency categories: <1%, 1–5% and 45–50%, using the two-step imputation method and the 1000 Genomes reference panel.

## Results

The concordance rates and the proportion of non-missing genotypes obtained with the four imputation strategies evaluated in this study are shown in Table 1. For this analysis, imputed genotypes with INFO scores lower than 0.9 were defined as missing genotypes. The concordance rate was used as a measure of the imputation accuracy and the proportion of non-missing genotypes as a measure of imputation efficacy. The concordance rate was consistently high (>98%) for all the imputation strategies, but there were differences between methods in imputation efficacy. Using the single-step strategy produced slightly higher concordance rates than the two-step strategy (e.g. 99.1% vs. 98.4% when using the HapMap phase II combined reference sample, respectively), but at the expense of a lower proportion of non-missing genotypes (85.5% vs. 90.1%, respectively). The inclusion of the HapMap phase III Mexican American sample as a reference sample, in addition to the HapMap phase II combined sample, produced a marginal improvement both in concordance rate and proportion of non-missing genotypes (99.4% vs. 99.1% for concordance, and 85.9% vs. 85.5% for the proportion of non-missing genotypes, using the single-step approach). For the two-step approach, using the 1,000 Genomes reference sample did not alter the concordance rate with respect to the HapMap phase II combined sample (98.4% for both datasets). However, the use of the 1000 Genomes panel produced a substantial increase in the proportion of non-missing genotypes (94.7% vs. 90.1%, respectively).

**Table 1:** Concordance rate and proportion of non-missing genotypes (using a score information threshold of 0.9) for chromosome 12 markers in the studied reference panels

**Reference Panel**	**IMPUTE Version**	**Concordance rate (%)**	**Proportion of non-missing genotypes (%)**
HapMap phase II combined	Version 1 (Single-step)	99.09	85.5
HapMap phase II combined + MXL	Version 1 (Single-step)	99.37	85.9
HapMap phase II combined	Version 2 (Two-steps: Phasing & Imputation)	98.40	90.1
1,000 Genomes Phase I (June 2011 release)	Version 2 (Two-steps: Phasing & Imputation)	98.44	94.7

**Figure 1:**
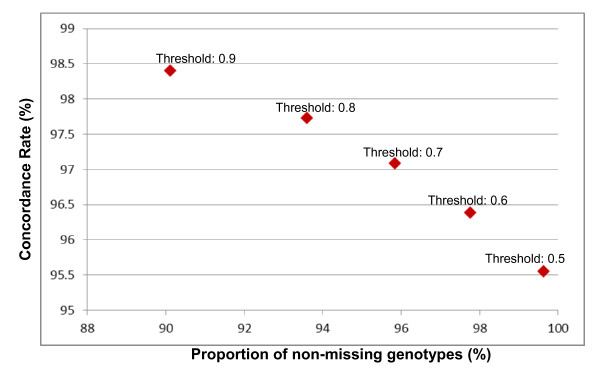
**Proportion of non-missing genotypes versus concordance rates using different INFO score thresholds.** This analysis was performed for the HapMap phase II combined reference sample based on the two-step imputation method.

Figure 1 illustrates the concordance rates and the proportion of non-missing genotypes obtained using various INFO score thresholds. This analysis provides an indication of how the selection of confidence thresholds affects the accuracy and efficacy of the imputations. We restricted this evaluation to the two-step protocol using the HapMap phase II combined sample. As expected, lowering the INFO score thresholds resulted in progressively reduced concordance rates and higher proportions of non-missing genotypes. Using a threshold of 0.9, the concordance rate was 98.4% and the proportion of non-missing genotypes 90.1%. Using a much less conservative threshold of 0.5, the concordance rate was still quite high (95.5%) and the proportion of non-missing genotypes went up to 99.6%.

**Figure 2:**
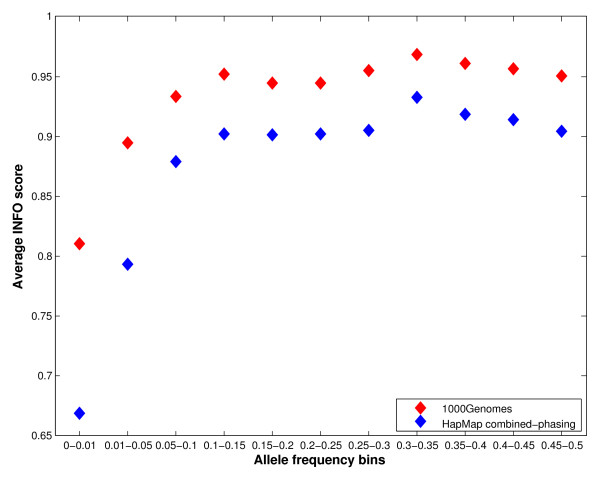
**Average INFO scores for different allele frequency bins of the HapMap phase II combined and the 1000 Genomes phase I reference panels.** The blue diamond represents the HapMap phase II combined panel and the red diamond represents the 1000 Genomes phase I panel (June 2011 release), based on the two-step imputation method.

**Figure 3:**
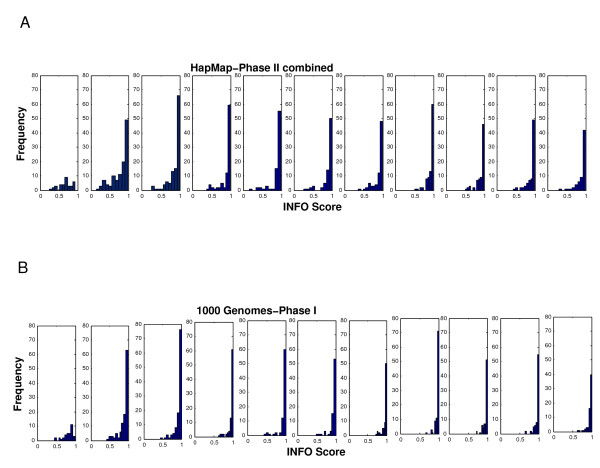
**Histogram showing the distribution of INFO scores within each allele frequency bin.** For the two-step imputation method based on the HapMap phase II combined reference panel. For the two-step imputation method based on the 1000 Genomes phase I (June 2011 release) reference panel.

**Figure 4:**
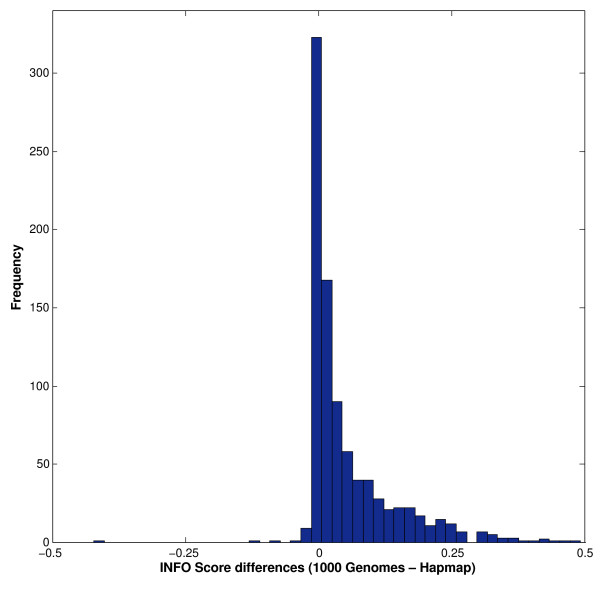
**Histogram showing the distribution of the differences in the INFO scores of HapMap phase II combined and the 1000 Genomes phase I reference panels.** The two-step imputation method based panels were only considered for this analysis.

**Figure 5:**
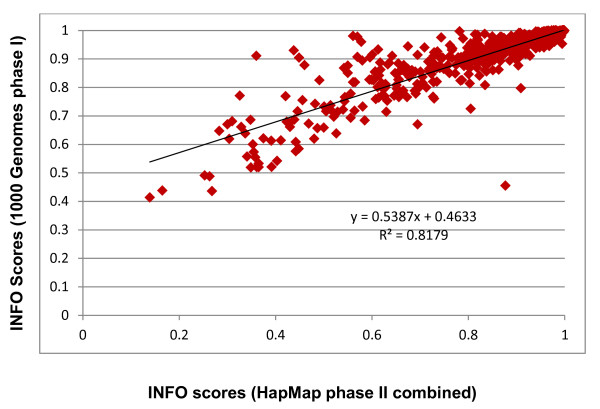
Correlation between the INFO scores for the two-step imputation method based on HapMap phase II combined and the 1000 Genomes phase I reference panels.

Figure 2 depicts the average INFO scores for different allele frequency bins, using the two-step imputation methods based on the HapMap phase II combined and the 1000 Genomes phase I (June 2011 release) reference panels. This figure provides information about imputation quality across the allele frequency spectrum, based on the two reference panels. The average INFO scores obtained for the 1000 Genomes panel are substantially higher, irrespective of the allele frequencies, than the HapMap phase II combined panel. It is also evident in the plot that rare alleles (frequencies < 5%) have considerably lower INFO scores than common alleles. In addition to average imputation qualities, it is also relevant to explore the distribution of INFO scores in each frequency bin. This is depicted in Figures 3A (for the HapMap Phase II combined reference sample) and 3B (for the 1000 Genomes phase I panel). These Figures show that for most frequency bins, the majority of the untyped SNPs have INFO scores higher than 0.9, with decreasing proportions of markers in the lower INFO score categories. However, for rare markers, particularly those with frequencies < 1%, the distribution is considerably wider, and the mode of the distribution does not correspond to the INFO score > 0.9, but to lower INFO score values. Additionally, the plots also demonstrate that using the 1000 Genomes sample as a reference sample shifts the distributions to the right in all the frequency bin categories. Markers imputed using the 1000 Genomes reference sample tend to have INFO scores higher than those imputed using the HapMap Phase II combined reference panel for all the frequency bins. This is also evident in Figure 4, which shows a histogram showing the distribution of the differences in INFO scores between the two-step imputation methods based on the 1000 Genomes Phase I and the HapMap Phase II combined reference samples. The correlation between the INFO scores of the two-step imputation methods based on the 1000 Genomes Phase I and the HapMap Phase II combined reference samples is shown in Figure 5. The INFO scores are highly correlated (r^2^ = 0.82): imputed markers with low INFO scores using the HapMap phase II combined panel also exhibit low INFO scores employing the 1000 Genomes phase I panel, although as described before, markers imputed with the 1000 Genomes panel showed higher INFO scores relative to those imputed with the HapMap sample.

We compared in more detail the imputation accuracy and efficacy for markers in the following allele frequency categories: <1%, 1–5% and 45–50%, using the two-step imputation method and the 1000 Genomes reference panel. For this analysis, instead of using the overall imputation concordance based on the three possible genotypes, we focused our attention on the concordance and missingness rates for the heterozygotes. The reason for employing this strategy is that an analysis based on overall imputation concordance may give misleading results for rare markers: the overall concordance rate may be high for these markers, but the concordance rates for heterozygotes and minor allele homozygotes may be much lower than the overall concordance rates. For the imputed markers in the 45–50% allele frequency bin, using an INFO threshold of 0.9, the concordance rate for the heterozygotes was 97.5% and the proportion of non-missing genotypes 90.2%. For the markers in the 1–5% bin, the concordance rate dropped to 85.4% and the proportion of non-missing genotypes to 85.1%. For rare markers (<1%), the drop was even more accused: the concordance rate was only 60.6% and the proportion of non-missing genotypes was 78.1%.

The results described above are based on markers located on chromosome 12. In order to evaluate the generalizability of these results, we also masked 5% of genotyped markers on chromosome 22, and on the HLA region, which spans approximately 5 megabases on chromosome 6 and has been under selective pressure in different population groups [31-33]. These analyses were carried out with the two-step imputation method using the HapMap and 1000 Genomes reference panels. For chromosome 22, using the HapMap reference panel, the concordance rate was 97.6%, and the proportion of non-missing genotypes 83.2%, and using the 1000 Genomes reference panel, the concordance rate was 97.3% and the proportion of non-missing genotypes 89.9%. For the HLA region, using the HapMap reference panel the concordance rate was 99.35% and the proportion of non-missing genotypes 97.4%, and with the 1000 Genomes reference panel the concordance rate was 99.5% and the proportion of non-missing genotypes 99.05%.

## Discussion

In recent years, imputation has become a key tool in the success of genome-wide association studies. Genotype imputation has proven to increase the power of genetic association studies, by boosting the number of SNPs to be tested for association and facilitating the detection of rare variants in addition to common variants [14,19,34,35]. Furthermore, imputation aids in fine-mapping studies of the disease-associated region thus increasing the chance of identifying additional candidate SNPs [36]. Finally, genotype imputation enables meta-analysis that combines results across studies based on different genotyping platforms [37,38]. This approach has been effective in identifying novel associations in different traits [39-44].

However, an important concern with respect to imputation lies in the selection of an appropriate reference panel. Most of the GWAS to date have been conducted in populations well represented by the available reference panels (e.g. European or East Asian populations), and used only one relevant reference population during the imputation process [6,43,45-47]. However, for populations that are phylogenetically distant from the samples present in the reference panels, the selection of a suitable reference panel for imputation becomes less clear. In this situation, differences in the pattern of LD between the study and reference populations may affect imputation accuracy. Different approaches have been suggested for this particular scenario. For example, Huang et al. [16] explored imputation accuracy in the samples of the HGDP-CEPH panel, which is a worldwide collection of individuals from different locations, using the HapMap II reference panels. The authors found that for most of the studied samples, mixtures from at least two HapMap reference samples maximized imputation accuracy [16]. Another study showed that using tag SNPs from all the HapMap reference populations combined captured common variation in African American, Latino and Hawaiian samples more effectively than tag SNPs obtained from the individual HapMap reference samples [48]. This ‘cosmopolitan’ approach to imputation, combining reference haplotypes from all the reference populations available, is the strategy currently recommended by the most widely used imputation packages, such as IMPUTE [19,20] and MACH [21].

African American and Hispanic/Latino populations have unique challenges for imputation. These populations are the result of recent admixture between continental groups (primarily European, Native American and West African populations) and admixture proportions show substantial geographic variation [49-51]. Several studies have evaluated imputation performance in recently admixed populations. In a recent GWAS of coronary heart disease and its risk factors in a large African American sample [52], a high imputation concordance (95.6%) was obtained when SNPs were imputed using a combined reference panel of haplotypes from the HapMap phase II CEU and YRI panels. In another study in African Americans [53], the highest imputation yield and coverage were attained using the two HapMap reference panels (CEU and YRI) separately and then merging the results. Another approach for imputation in African American populations has been recently suggested by Paşaniuc et al. (2011) [54]. This strategy, termed ‘local ancestry aware imputation’, uses local ancestry to guide the choice of reference haplotypes for imputation and shows marginal improvement in imputation accuracy in the admixed sample. However, this approach will be more difficult to implement in Hispanic/Latino populations, due to the lack of reference data for the relevant Native American parental populations, which is key to obtain accurate estimates of local ancestry. In the study by Huang et al. (2009), using combinations of two (European and East Asian) or three HapMap reference samples (East Asian, European and West African) produced the highest imputation accuracies (>95%) for two Native American samples (Pima and Maya) and a sample from Colombia [16]. A recent study [55] showed that, when performing imputation in a Hispanic sample from San Francisco with the program IMPUTE v2 and the HapMap II reference panel, using local haplotype weights based on a coalescent method provided lower error rates (7.8%) than using no weighting (8.9%), or a global weighting method based on empirical estimates of ancestry (9.0%) [56]. It is important to note that most of the aforementioned studies have used the HapMap II panel as the reference dataset for imputation. However, the recent progress of the 1000 Genomes project (http://www.1000genomes.org/) has provided the scientific community with much more complete reference panels, both in terms of the number of markers and the number of populations. Importantly, the reference databases are updated on a regular basis. For this reason, it is currently recommended to perform the imputation in two stages: pre-phasing the study genotypes to estimate haplotypes, and then imputing untyped genotypes in a separate run. This substantially reduces imputation time with respect to single-step approaches at the expense of a small loss in accuracy. An important advantage of this approach is that, as new reference data become available, it is only necessary to repeat the imputation step.

In this study, we evaluated the imputation performance of the widely used program IMPUTE in an admixed sample from Mexico City using different imputation strategies (single-step vs. two-step imputation) and reference panels (HapMap and 1000 Genomes). We have previously described that this sample primarily has Native American (62%) and European contributions (33%), with a low proportion of African ancestry (5%) [30]. Importantly, there are no Native American reference samples in the HapMap or 1000 Genomes datasets, so it is of relevance to test the relative imputation performance of these reference panels in the Mexican sample. In an analysis of imputed markers on chromosome 12, we observed that for this sample there are only minor differences in imputation accuracy between the single-step and two-step approaches (Table 1). The concordance rate of the single-step approach is only slightly higher than that of the two-step approach (99.1% vs. 98.4% when using the HapMap phase II combined reference sample, respectively). In contrast, the imputation efficacy (i.e. proportion of non-missing genotypes) was higher for the two-step than the single-step imputation approach (90.1% vs. 85.5%, respectively). Therefore, our study confirms the two-step approach as the preferable imputation strategy, because it provides flexibility and faster imputation times, while providing an overly similar imputation performance to the single-step approach.

As expected, we observed that adding the HapMap Phase III Mexican American sample from LA to the HapMap Phase II combined reference sample there were marginal increases in both accuracy (99.4% vs. 99.1%) and efficacy (85.9% vs. 85.5%) (Table 1). We also anticipated to find that reducing the threshold of the imputation confidence scores (the INFO score measures) when calling the imputed genotypes would result on lower imputation accuracy and higher proportions of non-missing genotypes. The reductions observed in imputation accuracy were relatively minor, from 98.4% with an INFO score threshold of 0.9 to 95.5% with an INFO score threshold of 0.5 (Figure 1). This relatively small reduction in overall imputation accuracy is primarily due to the fact that most genotypes (and markers) have very high INFO scores. Therefore, adding the relatively small percentage of genotypes with lower INFO scores (and lower concordance rates) does not produce a major shift in overall imputation accuracy. Of all masked markers on chromosome 12, 61.5% had INFO scores higher than 0.9, 15.4% had INFO scores between 0.8 and 0.9, 6.5% had INFO scores between 0.7 and 0.8, 6.2% had info scores between 0.6 and 0.7, 3.8% had INFO scores between 0.5 and 0.6, and 6.7% INFO scores lower than 0.5 (see also discussion below about the relationship of imputation efficacy and accuracy and allele frequency).

We also examined the potential improvement in imputation performance obtained with the recently available 1000 Genomes panel (June 2011 release), with respect to the HapMap panel, using the two-step imputation protocol. The 1000 Genomes panel is a much more comprehensive and powerful resource for imputation, comprising more than 37 million autosomal SNPs present in 1,094 individuals from different populations around the world. Here, we show that for the Mexican sample the major improvement associated with the use of the 1000 Genomes reference panel is the substantial increase in imputation efficacy, in addition to the larger number of imputed markers (Table 1). Genotype concordances were similar for both reference datasets (around 98.4%). However, imputations with the 1000 Genomes panel resulted in 94.7% of non-missing genotypes (employing an INFO score threshold of 0.9), in comparison with 90.1% for the HapMap phase II combined panel (using the same threshold). When the INFO scores are plotted for different allele frequency bins, either as an average (Figure 2) or as histograms of the individual scores (Figures 3A and 3B), it is evident that the confidence of the genotype calls is higher with the 1000 Genomes panel for all allele frequency categories. There is a high correlation between the INFO scores obtained with the 1000 Genomes and HapMap phase II reference panels (Figure 5), but the former are systematically higher than the latter (Figure 4).

The results described above are based on an analysis of markers on chromosome 12. An analysis of markers on chromosome 22 gives consistent results: The concordance rates using the HapMap phase II and 1000 Genomes reference panels are very similar (97.6% vs. 97.3%, respectively), but the proportion of non-missing genotypes is lower with the HapMap reference panel than with the 1000 Genomes panel (83.2%, and 89.9%, respectively). Interestingly, in the HLA region on chromosome 6, which spans approximately 5 megabases (29–34 Mb) and has shown signatures of natural selection in previous studies (31–33), both the imputation accuracy (concordance) and the imputation efficacy (proportion of non-missing genotypes) were higher than those observed for chromosomes 12 and 22. When analyzing locus ancestry with a panel of Ancestry Informative Markers in the sample from Mexico City (data not shown), we observed that in a broad region of chromosome 6, including the HLA loci, there was an excess of European ancestry with respect to the rest of the genome, in both type 2 diabetes patients and controls. This may be a potential explanation for the increased imputation accuracy and efficacy identified in the HLA region (i.e. both reference panels, HapMap and 1000 Genomes, have a good representation of European populations, but Native American populations are not well represented in these panels).

The imputation performance of the 1000 Genomes reference panel for rare variants is substantially better than that of the HapMap phase II panel. However, the average imputation confidence (INFO score) is considerably lower for rare variants than for common variants (Figures 2 and 3), irrespective of the reference panel. The rare alleles (<1%) present in the Mexican sample are not properly captured by any of the reference panels, in spite of the inclusion in the 1000 Genomes panel of dense data from another sample of Mexican ancestry from LA. This is also evident in a more detailed comparison of imputation accuracy and efficacy for heterozygotes in the following allele frequency categories: <1%, 1–5% and 45–50%. For common variants (45–50%), the imputation accuracy and efficacy were very high (>97% concordance and >90% non-missing genotypes). However, for rare variants (<1%), the proportion of missing genotypes was quite high (> 21%), and importantly, even for the genotypes with high INFO scores (>0.9), there was a large proportion of discordant calls (>39%). It is important to note that our analyses were based on markers from a commercial microarray (in order to minimize genotyping errors, the program PLINK was used to merge the genotype calls obtained with two genotyping algorithms: BRLMM-P and Birdseed), and it is not clear to which extent these findings can be extrapolated to other scenarios (e.g. sequencing data). However, our results highlight the need to be cautious with the interpretation of the results for rare variants in GWAS in Hispanic samples.

## Conclusions

We show that the program IMPUTE has an excellent imputation performance for common markers in an admixed sample from Mexico City, which has primarily Native American (62%) and European (33%) contributions. Genotype concordances for randomly masked markers are higher than 98.4% using different imputation strategies, in spite of the fact that no Native American samples are present in the HapMap and 1000 Genomes reference panels. In this sample, the best balance of imputation accuracy and efficiency was obtained with the 1,000 Genomes panel (genotype concordance 98.4% and proportion of non-missing genotypes 94.7%). However, not unexpectedly, rare alleles (frequencies <1%) are not captured efficiently by any of the available panels.

## Competing interests

The authors declare that they have no competing interests.

## Authors’ contributions

SK carried out the imputations with the program IMPUTE, analyzed the imputation results and wrote the manuscript. AVS, JP, JEP, JKR and MC recruited participants in Mexico City and coordinated the extraction of DNA. EJP coordinated the genotyping of the samples, conceived the study, and helped to draft the manuscript. All authors read and approved the final manuscript

## Pre-publication history

The pre-publication history for this paper can be accessed here:

http://www.biomedcentral.com/1755-8794/5/12/prepub

## References

[B1] Kruglyak L Prospects for whole-genome linkage disequilibrium mapping of common disease genes Nat Genet 1999 22 139 144 10.1038/9642 10369254

[B2] Sebastiani P Timofeev N Dworkis DA Perls TT Steinberg MH Genome-wide association studies and the genetic dissection of complex traits Am J Hematol 2009 84 504 515 10.1002/ajh.21440 19569043 PMC2895326

[B3] Kruglyak L The road to genome-wide association studies Nat Rev Genet 2008 9 314 318 18283274 10.1038/nrg2316

[B4] McCarthy MI Abecasis GR Cardon LR Goldstein DB Little J Ioannidis JP Hirschhorn JN Genome-wide association studies for complex traits: consensus, uncertainty and challenges Nat Rev Genet 2008 9 356 369 10.1038/nrg2344 18398418

[B5] Frazer KA Murray SS Schork NJ Topol EJ Human genetic variation and its contribution to complex traits Nat Rev Genet 2009 10 241 251 19293820 10.1038/nrg2554

[B6] The Wellcome Trust Case Control Consortium Genome-wide association study of 14,000 cases of seven common diseases and 3,000 shared controls Nature 2007 447 661 678 10.1038/nature05911 17554300 PMC2719288

[B7] Tsuchihashi Z Dracopoli NC Progress in high throughput SNP genotyping methods Pharmacogenomics J 2002 2 103 110 10.1038/sj.tpj.6500094 12049172

[B8] Low YL Wedrén S Liu J High-throughput genomic technology in research and clinical management of breast cancer Evolving landscape of genetic epidemiological studies. Breast Cancer Res 2006 8 209 10.1186/bcr1511 PMC1557740 16834767

[B9] Ku CS Kasiman K Chia KS John Wiley High-Throughput Single Nucleotide Polymorphisms Genotyping Technologies Encyclopedia of Life Sciences (ELS) 2009 Sons, Ltd, Chichester http://www.els.net [doi: 10.1002/9780470015902.a0021631]

[B10] Wang WY Barratt BJ Clayton DG Todd JA Genome-wide association studies: theoretical and practical concerns Nat Rev Genet 2005 6 109 118 10.1038/nrg1522 15716907

[B11] Barrett JC Cardon LR Evaluating coverage of genome-wide association studies Nat Genet 2006 38 659 662 10.1038/ng1801 16715099

[B12] Pe'er I de Bakker PI Maller J Yelensky R Altshuler D Daly MJ Evaluating and improving power in whole-genome association studies using fixed marker sets Nat Genet 2006 38 663 667 10.1038/ng1816 16715096

[B13] Donnelly P Progress and challenges in genome-wide association studies in humans Nature 2008 456 728 731 10.1038/nature07631 19079049

[B14] Marchini J Howie B Genotype imputation for genome-wide association studies Nat Rev Genet 2010 11 499 511 10.1038/nrg2796 20517342

[B15] Li Y Willer C Sanna S Abecasis G Genotype Imputation Annu Rev Genomics Hum Genet 2009 10 387 406 10.1146/annurev.genom.9.081307.164242 19715440 PMC2925172

[B16] Huang L Li Y Singleton AB Hardy JA Abecasis G Rosenberg NA Scheet P Genotype-imputation accuracy across worldwide human populations Am J Hum Genet 2009 84 2 235 250 10.1016/j.ajhg.2009.01.013 19215730 PMC2668016

[B17] Anderson CA Pettersson FH Barrett JC Evaluating the effects of imputation on the power, coverage, and cost efficiency of genome-wide SNP platforms Am J Hum Genet 2008 83 112 119 10.1016/j.ajhg.2008.06.008 18589396 PMC2443836

[B18] Almeida MA Oliveira PS Pereira TV Krieger JE Pereira AC An empirical evaluation of imputation accuracy for association statistics reveals increased type-I error rates in genome-wide associations BMC Genet 2011 12 10 21251252 10.1186/1471-2156-12-10 PMC3224203

[B19] Marchini J Howie B Myers S McVean G Donnelly P A new multipoint method for genome-wide association studies by imputation of genotypes Nat Genet 2007 39 906 913 10.1038/ng2088 17572673

[B20] Howie BN Donnelly P Marchini J A flexible and accurate genotype imputation method for the next generation of genome-wide association studies Plos Genet 2009 5 6 e1000529 10.1371/journal.pgen.1000529 19543373 PMC2689936

[B21] Li Y Willer CJ Ding J Scheet P Abecasis GR MaCH: using sequence and genotype data to estimate haplotypes and unobserved genotypes Genet Epidemiol 2010 34 816 834 10.1002/gepi.20533 21058334 PMC3175618

[B22] Browning SR Browning BL Rapid and accurate haplotype phasing and missing-data inference for whole-genome association studies by use of localized haplotype clustering Am J Hum Genet 2007 81 1084 1097 10.1086/521987 17924348 PMC2265661

[B23] Scheet P Stephens M A fast and flexible statistical model for large-scale population genotype data: applications to inferring missing genotypes and haplotypic phase Am J Hum Genet 2006 78 629 644 10.1086/502802 16532393 PMC1424677

[B24] Purcell S Neale B Todd-Brown K Thomas L Ferreira MA Bender D Maller J Sklar P de Bakker PI Daly MJ Sham PC PLINK: a tool set for whole-genome association and population-based linkage analyses Am J Hum Genet 2007 81 559 575 10.1086/519795 17701901 PMC1950838

[B25] Pei YF Li J Zhang L Papasian CJ Deng HW Analyses and comparison of accuracy of different genotype imputation methods PLoS One 2008 3 10 e3551 10.1371/journal.pone.0003551 18958166 PMC2569208

[B26] Browning SR Missing data imputation and haplotype phase inference for genome-wide association studies Hum Genet 2008 124 5 439 450 10.1007/s00439-008-0568-7 18850115 PMC2731769

[B27] Nothnagel M Ellinghaus D Schreiber S Krawczak M Franke A A comprehensive evaluation of SNP genotype imputation Hum Genet 2009 125 2 163 171 10.1007/s00439-008-0606-5 19089453

[B28] Hao K Chudin E McElwee J Schadt EE Accuracy of genome-wide imputation of untyped markers and impacts on statistical power for association studies BMC Genet 2009 10 27 19531258 10.1186/1471-2156-10-27 PMC2709633

[B29] Pei YF Zhang L Li J Deng HW Analyses and comparison of imputation-based association methods PLoS One 2010 5 5 e10827 10.1371/journal.pone.0010827 20520814 PMC2877082

[B30] Parra EJ Below JE Krithika S Valladares A Barta JL Cox NJ Hanis CL Wacher N Garcia-Mena J Hu P Shriver MD Diabetes Genetics Replication and Meta-analysis (DIAGRAM) Consortium, Kumate J, McKeigue PM, Escobedo J, Cruz M: Genome-wide association study of type 2 diabetes in a sample from Mexico City and a meta-analysis of a Mexican-American sample from Starr County, Texas Diabetologia 2011 54 8 2038 2046 10.1007/s00125-011-2172-y 21573907 PMC3818640

[B31] Bhatia G Patterson N Pasaniuc B Zaitlen N Genovese G Pollack S Mallick S Myers S Tandon A Spencer C Palmer CD Adeyemo AA Akylbekova EL Cupples LA Divers J Fornage M Kao WH Lange L Li M Musani S Mychaleckyj JC Ogunniyi A Papanicolaou G Rotimi CN Rotter JI Ruczinski I Salako B Siscovick DS Tayo BO Yang Q Genome-wide comparison of African-ancestry populations from CARe and other cohorts reveals signals of natural selection Am J Hum Genet 2011 89 3 368 381 10.1016/j.ajhg.2011.07.025 21907010 PMC3169818

[B32] Buhler S Sanchez-Mazas A HLA DNA sequence variation among human populations: molecular signatures of demographic and selective events PLoS One 2011 6 2 e14643 10.1371/journal.pone.0014643 21408106 PMC3051395

[B33] Albrechtsen A Moltke I Nielsen R Natural selection and the distribution of identity-by-descent in the human genome Genetics 2010 186 1 295 308 10.1534/genetics.110.113977 20592267 PMC2940294

[B34] Guan Y Stephens M Practical issues in imputation-based association mapping PLoS Genet 2008 4 12 e1000279 10.1371/journal.pgen.1000279 19057666 PMC2585794

[B35] Spencer CC Su Z Donnelly P Marchini J Designing genome-wide association studies: sample size, power, imputation, and the choice of genotyping chip PLoS Genet 2009 5 5 e1000477 10.1371/journal.pgen.1000477 19492015 PMC2688469

[B36] Liu JZ Tozzi F Waterworth DM Pillai SG Muglia P Middleton L Berrettini W Knouff CW Yuan X Waeber G Vollenweider P Preisig M Wareham NJ Zhao JH Loos RJ Barroso I Khaw KT Grundy S Barter P Mahley R Kesaniemi A McPherson R Vincent JB Strauss J Kennedy JL Farmer A McGuffin P Day R Matthews K Bakke P Wellcome Trust Case Control Consortium, Mooser V, Francks C, Marchini J: Meta-analysis and imputation refines the association of 15q25 with smoking quantity Nat Genet 2010 42 5 436 40 10.1038/ng.572 20418889 PMC3612983

[B37] de Bakker PI Ferreira MA Jia X Neale BM Raychaudhuri S Voight BF Practical aspects of imputation-driven meta-analysis of genome-wide association studies Hum Mol Genet 2008 17 R2 R122 128 10.1093/hmg/ddn288 18852200 PMC2782358

[B38] Zeggini E Ioannidis JP Meta-analysis in genome-wide association studies Pharmacogenomics 2009 10 2 191 201 10.2217/14622416.10.2.191 19207020 PMC2695132

[B39] Cooper JD Smyth DJ Smiles AM Plagnol V Walker NM Allen JE Downes K Barrett JC Healy BC Mychaleckyj JC Warram JH Todd JA Meta-analysis of genome-wide association study data identifies additional type 1 diabetes risk loci Nat Genet 2008 40 12 1399 1401 10.1038/ng.249 18978792 PMC2635556

[B40] De Jager PL Jia X Wang J de Bakker PI Ottoboni L Aggarwal NT Piccio L Raychaudhuri S Tran D Aubin C Briskin R Romano S International MS Genetics Consortium Baranzini SE McCauley JL Pericak-Vance MA Haines JL Gibson RA Naeglin Y Uitdehaag B Matthews PM Kappos L Polman C McArdle WL Strachan DP Evans D Cross AH Daly MJ Compston A Sawcer SJ Meta-analysis of genome scans and replication identify CD6, IRF8 and TNFRSF1A as new multiple sclerosis susceptibility loci Nat Genet 2009 41 7 776 782 10.1038/ng.401 19525953 PMC2757648

[B41] Houlston RS Cheadle J Dobbins SE Tenesa A Jones AM Howarth K Spain SL Broderick P Domingo E Farrington S Prendergast JG Pittman AM Theodoratou E Smith CG Olver B Walther A Barnetson RA Churchman M Jaeger EE Penegar S Barclay E Martin L Gorman M Mager R Johnstone E Midgley R Niittymäki I Tuupanen S Colley J Idziaszczyk S Meta-analysis of three genome-wide association studies identifies susceptibility loci for colorectal cancer at 1q41, 3q26.2, 12q13.13 and 20q13.33 Nat Genet 2010 42 11 973 977 10.1038/ng.670 20972440 PMC5098601

[B42] International Parkinson Disease Genomics Consortium Nalls MA Plagnol V Hernandez DG Sharma M Sheerin UM Saad M Simón-Sánchez J Schulte C Lesage S Sveinbjörnsdóttir S Stefánsson K Martinez M Hardy J Heutink P Brice A Gasser T Singleton AB Wood NW Imputation of sequence variants for identification of genetic risks for Parkinson's disease: a meta-analysis of genome-wide association studies Lancet 2011 377 9766 641 649 21292315 10.1016/S0140-6736(10)62345-8 PMC3696507

[B43] Strawbridge RJ Dupuis J Prokopenko I Barker A Ahlqvist E Rybin D Petrie JR Travers ME Bouatia-Naji N Dimas AS Nica A Wheeler E Chen H Voight BF Taneera J Kanoni S Peden JF Turrini F Gustafsson S Zabena C Almgren P Barker DJ Barnes D Dennison EM Eriksson JG Eriksson P Eury E Folkersen L Fox CS Frayling TM Genome-wide association identifies nine common variants associated with fasting proinsulin levels and provides new insights into the pathophysiology of type 2 diabetes Diabetes 2011 60 10 2624 2634 10.2337/db11-0415 21873549 PMC3178302

[B44] Zeggini E Scott LJ Saxena R Voight BF Marchini JL Hu T de Bakker PI Abecasis GR Almgren P Andersen G Ardlie K Boström KB Bergman RN Bonnycastle LL Borch-Johnsen K Burtt NP Chen H Chines PS Daly MJ Deodhar P Ding CJ Doney AS Duren WL Elliott KS Erdos MR Frayling TM Freathy RM Gianniny L Grallert H Grarup N Meta-analysis of genome-wide association data and large-scale replication identifies additional susceptibility loci for type 2 diabetes Nat Genet 2008 40 5 638 645 10.1038/ng.120 18372903 PMC2672416

[B45] Scott LJ Mohlke KL Bonnycastle LL Willer CJ Li Y Duren WL Erdos MR Stringham HM Chines PS Jackson AU Prokunina-Olsson L Ding CJ Swift AJ Narisu N Hu T Pruim R Xiao R Li XY Conneely KN Riebow NL Sprau AG Tong M White PP Hetrick KN Barnhart MW Bark CW Goldstein JL Watkins L Xiang F Saramies J A genome-wide association study of type 2 diabetes in Finns detects multiple susceptibility variants Science 2007 316 5829 1341 1345 10.1126/science.1142382 17463248 PMC3214617

[B46] Kato N Takeuchi F Tabara Y Kelly TN Go MJ Sim X Tay WT Chen CH Zhang Y Yamamoto K Katsuya T Yokota M Kim YJ Ong RT Nabika T Gu D Chang LC Kokubo Y Huang W Ohnaka K Yamori Y Nakashima E Jaquish CE Lee JY Seielstad M Isono M Hixson JE Chen YT Miki T Zhou X Meta-analysis of genome-wide association studies identifies common variants associated with blood pressure variation in east Asians Nat Genet 2011 43 6 531 8 10.1038/ng.834 21572416 PMC3158568

[B47] Kim YJ Go MJ Hu C Hong CB Kim YK Lee JY Hwang JY Oh JH Kim DJ Kim NH Kim S Hong EJ Kim JH Min H Kim Y Zhang R Jia W Okada Y Takahashi A Kubo M Tanaka T Kamatani N Matsuda K Consortium MAGIC Park T Oh B Kimm K Kang D Shin C Cho NH Large-scale genome-wide association studies in East Asians identify new genetic loci influencing metabolic traits Nat Genet 2011 43 10 990 995 10.1038/ng.939 21909109

[B48] de Bakker PI Burtt NP Graham RR Guiducci C Yelensky R Drake JA Bersaglieri T Penney KL Butler J Young S Onofrio RC Lyon HN Stram DO Haiman CA Freedman ML Zhu X Cooper R Groop L Kolonel LN Henderson BE Daly MJ Hirschhorn JN Altshuler D Transferability of tag SNPs in genetic association studies in multiple populations Nat Genet 2006 38 11 1298 1303 10.1038/ng1899 17057720

[B49] Parra EJ Marcini A Akey J Martinson J Batzer MA Cooper R Forrester T Allison DB Deka R Ferrell RE Shriver MD Estimating African American admixture proportions by use of population-specific alleles Am J Hum Genet 1998 63 6 1839 1851 10.1086/302148 9837836 PMC1377655

[B50] Wang S Ray N Rojas W Parra MV Bedoya G Gallo C Poletti G Mazzotti G Hill K Hurtado AM Camrena B Nicolini H Klitz W Barrantes R Molina JA Freimer NB Bortolini MC Salzano FM Petzl-Erler ML Tsuneto LT Dipierri JE Alfaro EL Bailliet G Bianchi NO Llop E Rothhammer F Excoffier L Ruiz-Linares A Geographic patterns of genome admixture in Latin American Mestizos PLoS Genet 2008 4 3 e1000037 10.1371/journal.pgen.1000037 18369456 PMC2265669

[B51] Galanter JM Fernandez-Lopez JC Gignoux CR Barnholtz-Sloan J Fernandez-Rozadilla C Via M Hidalgo-Miranda A Contreras AV Figueroa LU Raska P Jimenez-Sanchez G Silva Zolezzi I Torres M Ponte CR Ruiz Y Salas A Nguyen E Eng C Borjas L Zabala W Barreto G Rondón González F Ibarra A Taboada P Porras L Moreno F Bigham A Gutierrez G Brutsaert T León-Velarde F for the LACE Consortium. Development of a Panel of Genome-Wide Ancestry Informative Markers to Study Admixture Throughout the Americas PLoS Genet 2012 8 3 1002554 10.1371/journal.pgen.1002554 PMC3297575 22412386

[B52] Lettre G Palmer CD Young T Ejebe KG Allayee H Benjamin EJ Bennett F Bowden DW Chakravarti A Dreisbach A Farlow DN Folsom AR Fornage M Forrester T Fox E Haiman CA Hartiala J Harris TB Hazen SL Heckbert SR Henderson BE Hirschhorn JN Keating BJ Kritchevsky SB Larkin E Li M Rudock ME McKenzie CA Meigs JB Meng YA Genome-wide association study of coronary heart disease and its risk factors in 8,090 African Americans: the NHLBI CARe Project PLoS Genet 2011 7 2 e1001300 10.1371/journal.pgen.1001300 21347282 PMC3037413

[B53] Shriner D Adeyemo A Chen G Rotimi CN Practical considerations for imputation of untyped markers in admixed populations Genet Epidemiol 2010 34 3 258 265 19918757 10.1002/gepi.20457 PMC2912698

[B54] Paşaniuc B Zaitlen N Lettre G Chen GK Tandon A Kao WH Ruczinski I Fornage M Siscovick DS Zhu X Larkin E Lange LA Cupples LA Yang Q Akylbekova EL Musani SK Divers J Mychaleckyj J Li M Papanicolaou GJ Millikan RC Ambrosone CB John EM Bernstein L Zheng W Hu JJ Ziegler RG Nyante SJ Bandera EV Ingles SA Enhanced statistical tests for GWAS in admixed populations: assessment using African Americans from CARe and a Breast Cancer Consortium PLoS Genet 2011 7 4 e1001371 10.1371/journal.pgen.1001371 21541012 PMC3080860

[B55] Paşaniuc B Avinery R Gur T Skibola CF Bracci PM Halperin E A generic coalescent-based framework for the selection of a reference panel for imputation Genet Epidemiol 2010 34 8 773 782 10.1002/gepi.20505 21058333 PMC3876740

[B56] Egyud MR Gajdos ZK Butler JL Tischfield S Le Marchand L Kolonel LN Haiman CA Henderson BE Hirschhorn JN Use of weighted reference panels based on empirical estimates of ancestry for capturing untyped variation Hum Genet 2009 125 3 295 303 10.1007/s00439-009-0627-8 19184111 PMC3126674

